# Genomic landscape of complete *Acinetobacter* phages: clustering, core-shell genes, and synteny insights

**DOI:** 10.3389/fmicb.2026.1720092

**Published:** 2026-01-28

**Authors:** Sean Jia Le Pang, Soon Keong Wee, Eric Peng Huat Yap

**Affiliations:** 1 Interdisciplinary Graduate Programme, Institute for Digital Molecular Analytics and Science, Nanyang Technological University, Singapore, Singapore; 2 Institute for Digital Molecular Analytics and Science, Nanyang Technological University, Singapore, Singapore; 3 Lee Kong Chian School of Medicine, Nanyang Technological University, Singapore, Singapore

**Keywords:** *Acinetobacter*, bacteriophage, comparative genomics, endolysins, phage engineering, phage therapy, proteomics

## Abstract

**Introduction:**

*Acinetobacter baumannii* is a major clinical threat due to its multidrug resistance and frequent involvement in nosocomial outbreaks. Bacteriophage therapy offers a targeted alternative, but its success depends on a deep understanding of phage genomics and proteomics. This study aims to cluster the current database of *Acinetobacter* phages, identify phage clusters with potential for therapeutic applications and highlight proteins that may be valuable for phage engineering.

**Methods:**

A total of 250 publicly available complete *Acinetobacter* phage genomes were downloaded from NCBI database. The genomes were grouped into clusters using PhamClust. A phylogenetic tree using the terminase large subunit and the portal protein was charted. Gene synteny analysis was conducted using Clinker, while the protein family output from PhaMMseqs was utilized to investigate both broadly conserved and cluster-specific phams.

**Results:**

*Acinetobacter* phages were classified into 12 clusters, including the newly identified cluster 10. While the terminase large subunit and portal protein proved useful for cluster-level grouping, they were insufficient for resolving finer phylogenetic relationships. Three conserved enzymes, endolysins, a DNA helicase, and an HNH homing endonuclease, were found across multiple clusters, alongside a diverse array of cluster-specific phams.

**Discussion:**

Cluster 10 was found to contain three phages with exceptionally broad host ranges, highlighting its strong potential for therapeutic development. Additionally, the three conserved enzymes shared across multiple clusters, especially the endolysins, may serve as valuable tools for phage engineering due to their broad conservation. The cluster-specific phams offer biological insights and may also form the basis for developing cluster-specific primers or molecular diagnostics. Collectively, these findings deepen our understanding of *Acinetobacter* phage diversity and point toward new avenues for advancing phage-based therapies and diagnostics.

## 1 Introduction

### 1.1 Global context of *Acinetobacter baumannii* and phage therapy

*Acinetobacter baumannii* is a gram-negative bacteria that has emerged as a formidable nosocomial threat worldwide, frequently causing diverse infections such as pneumonia, septicemia, meningitis, urinary tract and wound infections among critically ill patients in hospital settings (Castillo-Ramírez, 2023; Moubareck and Halat, 2020). This pathogen presents a significant global burden, with up to 1.4 million cases reported annually (Cain and Hamidian, 2023). The economic burden of *A. baumannii* infections is substantial, with per-infection costs ranging from tens to over a hundred thousand U. S. dollars, leading to annual national expenditures in the hundreds of millions to billions in the United States of America alone (Nelson et al., 2016).

*A. baumannii* is classified among the ESKAPE pathogens namely, *Enterococcus faecium*, *Staphylococcus aureus*, *Klebsiella pneumoniae*, *Pseudomonas aeruginosa*, and *Enterobacter* species which are well-known for acquiring antibiotics resistance rendering them powerless (Rice, 2008). Given that carbapenems are among humanity’s last-line antibiotics (Bradley et al., 1999), the urgency to combat the carbapenem resistant *A. baumannii* strains is heightened. In 2024, carbapenem-resistant *A. baumannii* was formally assigned a critical priority level by the WHO for the development of new control, treatment, and prevention measures (WHO Bacterial Priority Pathogens List, 2024). The extensive drug resistance of *A. baumannii* stems from multiple resistance determinants, including enzymatic inactivation, target modification, and efflux pumps, whose rapid spread is driven by mobile genetic elements (MGEs) such as plasmids and resistance islands (Scoffone et al., 2025).

Phage therapy offers a compelling strategy against extensively drug-resistant *Acinetobacter baumannii* (XDRAB) because bacteriophages operate independently of antibiotic resistance mechanisms (Qu et al., 2024). A primary advantage is Phage-Antibiotic Synergy (PAS), demonstrated when the lytic Phage Indie combined with ceftazidime achieved superior bactericidal effects *in vitro*. This synergy was effective in resensitizing resistant *A. baumannii* strains to the antibiotic, overcoming initial phage resistance, and allowing for treatment at lower doses. Furthermore, phages selected for therapy, such as phage Indie, can be safety-characterized to ensure the absence of lysogenic, virulence, or antibiotic resistance genes (Orozco-Ochoa et al., 2025). In addition, combined inhaled phage therapy has been associated with patient stabilization and showed no severe adverse reactions. Limitations of phage therapy includes the critical issue of phage resistance, observed in clinical cases where the phage lost significant lytic activity during extended treatment (Qu et al., 2024). Additionally, the effectiveness of combination therapy is also highly specific. While Phage Indie showed synergy with ceftazidime, it had an antagonistic effect with piperacillin-tazobactam (Orozco-Ochoa et al., 2025). Furthermore, inhaled phages pose biodistribution and safety concerns: phage DNA was detected in the bloodstream and appeared to accumulate in the intestines, resulting in significant alterations in gut microbiota composition. Additional clinical trials are necessary to fully assess the safety and implications of phage dissemination and its impact on intestinal microecology.

Despite the limitations, phage therapy and research is currently undergoing “a well-deserved rebirth” amid escalating resistance, re-emerged as a precision treatment, leveraging viruses that can destroy bacteria no longer susceptible to conventional antibiotics (Altamirano et al., 2021; Maasilta, 2025; Lin et al., 2017).

### 1.2 Prior work on phage clustering and core genomes

To unlock the full potential of phage therapy, reducing risks and reaping the greatest benefits, understanding phage genomics is indispensable. This could expand our toolkit for phage engineering and understanding which phage genes may be undesirable for therapy could help improve safety profiles. Our environment holds an immense reservoir of phages, researchers are constantly discovering and sequencing novel ones (Ranveer et al., 2024).

Earlier work on *A. baumannii* phage genomics demonstrated that the 37 complete *Acinetobacter* phage genomes segregate into six discrete clusters and two genomic singletons based on whole-genome dot-plots and average nucleotide identity (Turner et al., 2018). Proteomic clustering using OrthoMCL grouped 4,067 encoded proteins into 737 orthologous groups and 974 orphan proteins, with 56.6% of proteins annotated as hypothetical (Turner et al., 2018). However, only 42 *Acinetobacter* phage genomes were deposited in NCBI GenBank at the time, of which 37 were analyzed, meaning true diversity was likely under-sampled and cluster boundaries provisional. Isolates overwhelmingly belonged to *Caudovirales*, with only one ssRNA *Levivirus* and no filamentous *Inoviridae*, suggesting an incomplete view of *Acinetobacter* phage families. Furthermore, over half of the 4,067 encoded proteins lacked functional annotation, reflecting “genetic dark matter” in phage genomes that can only be resolved with more isolates and experimental validation.

Building on Turner et al., Oliveira and co-workers expanded the dataset to 139 publicly available *Acinetobacter* phage genomes as of 21 January 2021, re-annotating all sequences with Prokka against a custom *Caudovirales* database (Oliveira et al., 2022). They combined nucleotide and protein clustering with network and pan-genome analyses to delineate *Acinetobacter* phage taxonomy and evolutionary relationships.

Their study expanded from 37 to 139 genomes, enhancing cluster resolution and discovery of 5 proposed rare subfamilies (Oliveira et al., 2022). The use of integrated vConTACT2 and INPHARED pipelines allowed detection of 8 subfamily-level clusters and 46 genera, including eight singletons, which improves taxonomic granularity beyond pairwise ANI and OrthoMCL alone.

As the number of sequenced phages expands, a more sensitive, scalable clustering framework is needed to reveal truly novel lineages and test the stability of existing clusters against deeper sampling. Moreover, past *Acinetobacter* phage studies did not systematically mine these datasets for universally conserved proteins, prime candidates for phage engineering or for cluster-specific phamilies that could illuminate the distinct infection and replication strategies of each lineage. Addressing these gaps can be essential for translating comparative genomics into actionable tools for therapeutic development against *Acinetobacter* infections.

### 1.3 Scope and aims

In this study, we leveraged an expanded dataset of complete *Acinetobacter* phage genomes nearly double that of previous comparative analyses to generate a comprehensive, protein-family-based framework for phage classification and functional insight. We aim to systematically characterize and cluster *Acinetobacter* phages and uncover any previously unrecognized lineages; to quantify the evolutionary relationships among clusters; to pinpoint conserved “core” proteins and machineries within each group; and to identify phamilies that bridge multiple clusters, illuminating shared functional modules and potential targets for broad-spectrum phage engineering.

## 2 Materials and methods

All available protein sequences for complete *Acinetobacter* phages were retrieved from the NCBI Virus portal on 11 February, 2025. First, the NCBI Virus database was queried for “complete” nucleotide entries, and the host filter was set to Taxonomy ID 469 (*Acinetobacter* spp.). Protein sequences were downloaded in FASTA format, yielding a single multi-FASTA file containing 30,824 protein records derived from 250 distinct phage genomes.

To enable genome-by-genome analyses, this annotated multi-FASTA file was parsed and processed with a custom Python script (Supplementary material): it parsed each record’s header to identify its source genome accession, grouped sequences accordingly, and wrote one FASTA file per phage genome. These per-genome FASTA files served as the input for subsequent clustering and comparative analyses via PhaMMseqs version 1.0.4 (Gauthier et al., 2022) and PhamClust version 1.3.3 (Gauthier and Hatfull, 2023).

### 2.1 Clustering of phage genomes using PhamClust

We leveraged PhamClust, a scalable, gene-family-based clustering tool to delineate relationships among 250 *Acinetobacter* phage genomes. PhamClust integrates PhaMMseqs for phamilies (“phams”) assembly and computes a Proteomic Equivalence Quotient (PEQ) between every genome pair by averaging pairwise amino-acid identity across shared phams for genome-wide similarity (Gauthier and Hatfull, 2023).

We first generated phamilies of related proteins using PhaMMseqs version 1.0.4 (Gauthier et al., 2022; Gauthier and Hatfull, 2024). Multi-FASTA files each containing all proteins from a single phage genome were processed through MMseqs2’s sequence–sequence clustering through default settings (30% minimum identity, 80% coverage) to group proteins into preliminary clusters (phams). Profile-sequence alignments were performed to refine pham boundaries, ensuring that divergent homologs were correctly assigned. Resulting phams were exported as separate FASTA alignments and a genome pham presence/absence TSV, which served as input for PhamClust version 1.3.3. Phamilies were manually annotated by examining the top five gene annotations within each pham submitted by the depositors and assigning a consensus function based on the majority annotation. In instances where the majority was labeled as hypothetical proteins, the non-hypothetical annotations, if present, were selected to provide a more comprehensive functional representation. In addition, PhaMMseqs generated a pangenome summary table classifying phams into core, soft-core, shell, and cloud gene categories.

Next, using the pham presence/absence TSV and PEQ matrix, PhamClust executed a two-step clustering strategy using default parameters (Gauthier and Hatfull, 2023). First, genomes were grouped at a PEQ cutoff of 25% to establish broad clusters; second, each cluster was recursively subdivided at a higher PEQ threshold (50%) to resolve subclusters.

### 2.2 Phylogenetic analysis

To validate our proteome-based clusters, we reconstructed phylogenetic trees using two hallmark genes, the terminase large subunit and the portal protein. Both genes are universally present as single-copy, essential components of *Caudovirales* and exhibit sufficient sequence conservation to resolve deep and shallow evolutionary relationships (de Jonge et al., 2024).

Amino-acid sequences for each marker were extracted using PhaBOX version 2.0 and concatenated before alignment using Clustal Omega within the Jalview version 2.11.4.1 workbench (Sievers and Higgins, 2014; Waterhouse et al., 2009). Alignment using Clustal Omega was executed with default settings (35% identity threshold; 80% coverage) and subsequently used to compute a Newick-formatted neighbor joining phylogenetic tree for each marker. The phylogeny tree was visualized and annotated in Interactive Tree Of Life (iTOL) version 6.9.1 web server (Letunic and Bork, 2021, 2024).

### 2.3 PhaBOX

To contextualize our proteomic clusters with functional and taxonomic information, we applied the end-to-end workflow of the PhaBOX v2 web server (Shang et al., 2023) to all 250 *Acinetobacter* phage genomes.

PhaBOX utilized PhaTYP for lifestyle prediction and PhaGCN for taxonomic classification. PhaTYP employs a machine learning model and is able to achieve high accuracy for predicting lifestyles (Shang et al., 2022). PhaGCN integrates a graph convolutional network combining sequence-based features and gene-sharing networks to predict taxonomy per ICTV standards (Shang, 2021).

### 2.4 Gene synteny analyses using clinker

To explore genomic organization and infer functional modules related to phage infection and lysis, we employed Clinker (Gilchrist and Chooi, 2021) to generate comparative gene-synteny maps both between and within our PhamClust-defined clusters.

For between-cluster synteny comparisons, we randomly selected one representative genome from each PhamClust derived cluster and ran Clinker with its default settings to identify conserved gene blocks across distantly related phages. For within-cluster analysis of Cluster 10 the same default setting was run for all 3 respective phages.

All bioinformatics tools were run using default parameters unless otherwise stated.

## 3 Results

### 3.1 *Acinetobacter* phages are highly diverse with 12 proteomic clusters

**Figure 1:**
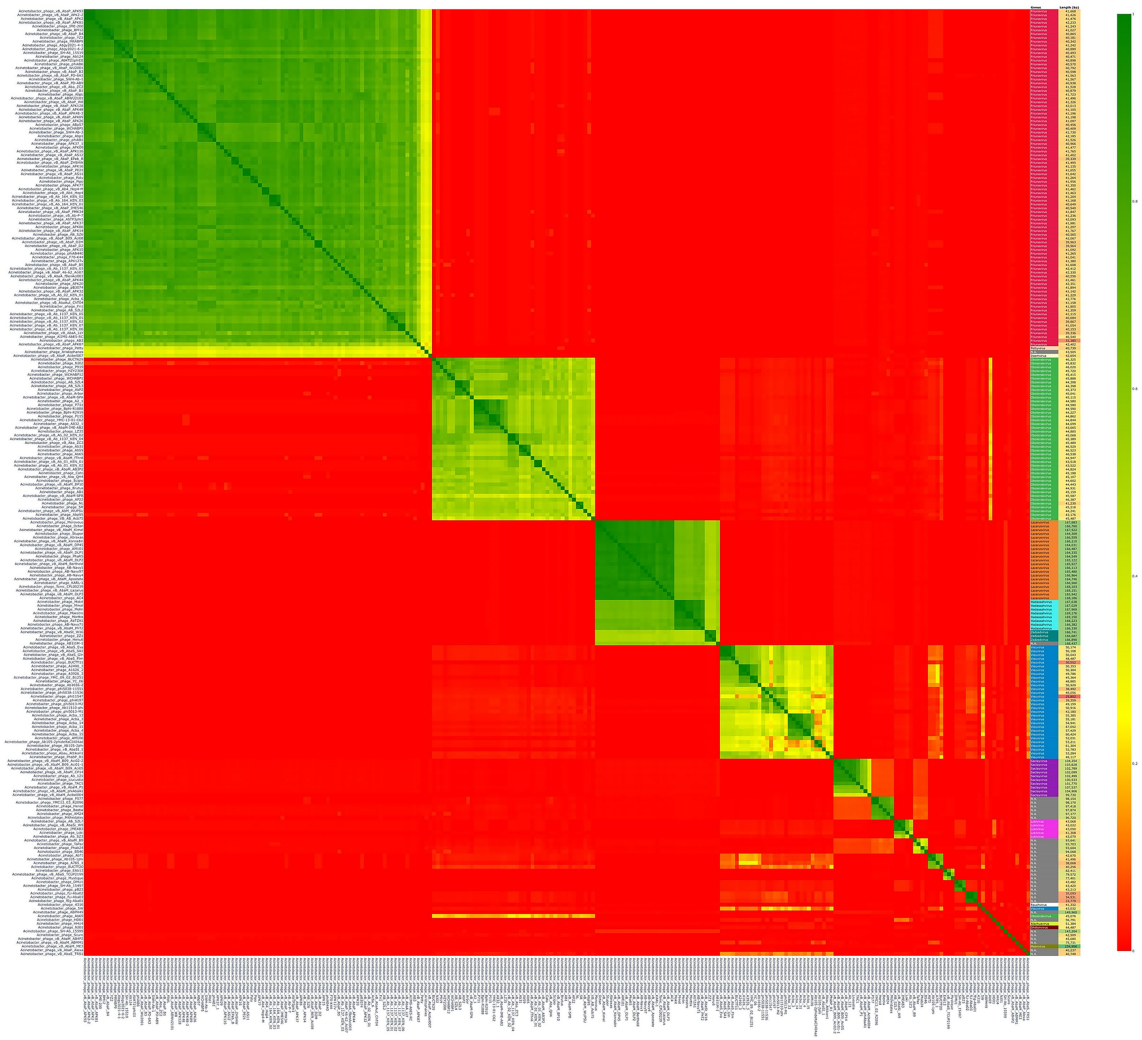
Proteomic Equivalence Quotient (PEQ) heatmap of 250 *Acinetobacter* phage genomes clustered by PhamClust. Pairwise PEQ values (0–100%) are displayed as a color gradient from red (no similarity) to yellow (slight similarity) to green (high similarity). Phage genomes are ordered along both axes by their PhamClust cluster assignment, with the 12 major clusters shown from largest (upper-left block) to smallest (lower-right blocks), followed by 14 singleton genomes. The ICTV-predicted genus and genome length are annotated along the right side of the heatmap.

A total of 250 *Acinetobacter* phages were clustered based on shared protein families into 12 major clusters ordered from largest to smallest, followed by 14 singletons (Figure 1).

Overall, the clear “block-diagonal” pattern observed in Figure 1 confirms that PhamClust robustly partitions the 250 *Acinetobacter* phages into biologically meaningful groups, while the extensive red regions attest to the extraordinary proteomic diversity across the phage population.

Next, to assess how tightly phages group within each cluster and how distinct each cluster is from its neighbors, we examined the pairwise PEQ values to quantify both intra-cluster cohesion and inter-cluster relatedness. The PhamClust heatmap (Figure 1) and cluster summary (Supplementary Table S1) reveal 12 discrete proteomic clusters, ranging from 92 phages in Cluster 1 down to three phages in Clusters 10–12, plus 14 singletons. Within-cluster PEQ values exceed 60–70%, indicating strong core pham conservation, while inter-cluster values typically fall below 10%. The presence of partial shell-gene sharing and hints at evolutionary proximity or horizontal gene flow. Clusters 4, 9 and 2 singletons share a small set of proteins, and Clusters 5, 6, and 8 exhibit overlaps with PEQ values exceeding 30% revealing inter cluster homology. Cluster 4 exhibits the least intra-cluster cohesion containing the most proteomically diverse phages.

Then, we characterized each cluster by several key parameters, the average genome length, the predominant lifecycle strategy (lytic versus temperate), and any existing ICTV-recognized genera assignments. The summaries of each cluster’s membership, mean genome length, relative standard error (RSE), PhaBOX-derived lifestyle breakdown, and ICTV genus annotations were noted (Supplementary Table S1). PhaBOX’s outputs can be found in Supplementary Table S2.

The phage genome lengths cover a wide range from 34,601 bp to 166,600 bp. All 12 clusters exhibit exceptionally low relative standard errors (RSE < 4%), indicating highly consistent average genome lengths within each group. Clusters 4 and 9, which contain mixtures of virulent and temperate phages, also showed the highest RSE values of 3.05 and 2.10%, respectively. Additionally, based on PEQ values (Figure 1), Cluster 4 has the greatest proteomic variability within the cluster with hints of subclusters. We also observed that two distinct groups of established ICTV genera clustered together. The first group comprised of the genera *Daemvirus*, *Friunavirus*, and *Pettyvirus*, while the second included *Hadassahvirus*, *Lazarusvirus*, and *Zedzedvirus*. Clusters 6 and 8 to 12 lack current ICTV genus assignments and represent novel genera.

Our work substantially extends the comparative framework established by Oliveira et al. (2022) through a nearly twofold increase in the number of phages in most clusters. In addition to the previously identified clusters, we resolved an entirely new cluster, known as Cluster 10 in this study, as well as 14 singleton genomes.

### 3.2 Concatenated terminase large-subunit and portal-protein sequences delineate phylogeny clustering

To evaluate whether rapid, marker-gene phylogenies can stand in for comprehensive proteome clustering, and to gauge inter-cluster relatedness, we compared phylogenetic trees built from the terminase large-subunit and portal proteins against our PhamClust results.

**Figure 2:**
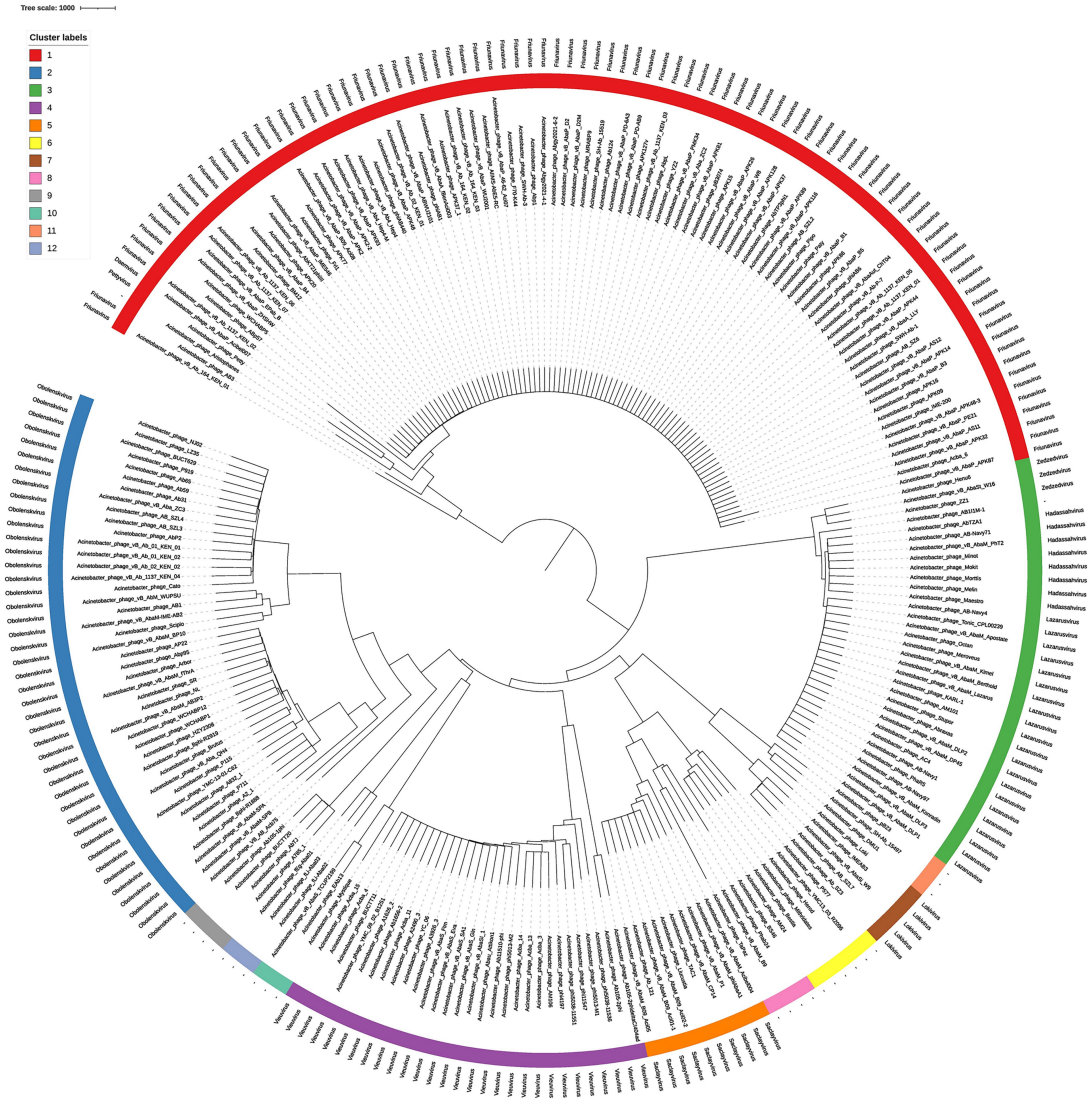
Neighbor joining phylogenetic tree of 250 *Acinetobacter* phage genomes based on concatenated terminase large-subunit and portal-protein sequences. Each tip is color-coded by PhamClust cluster, cluster labels are annotated as shown in the legend box on the left.

The concatenated terminase large-subunit and portal-protein phylogeny (Figure 2) exhibits near-perfect concordance with our PhamClust proteome clusters validating the specificity of the clusters. Additionally, tree topology reveals clusters that are phylogenetically close. Clusters 7 and 11 and Clusters 5, 6, and 8 emerge from a common branch in the phylogenetic tree, indicating they could potentially share a substantial subset of phamilies.

### 3.3 Gene synteny analysis between clusters

**Figure 3:**
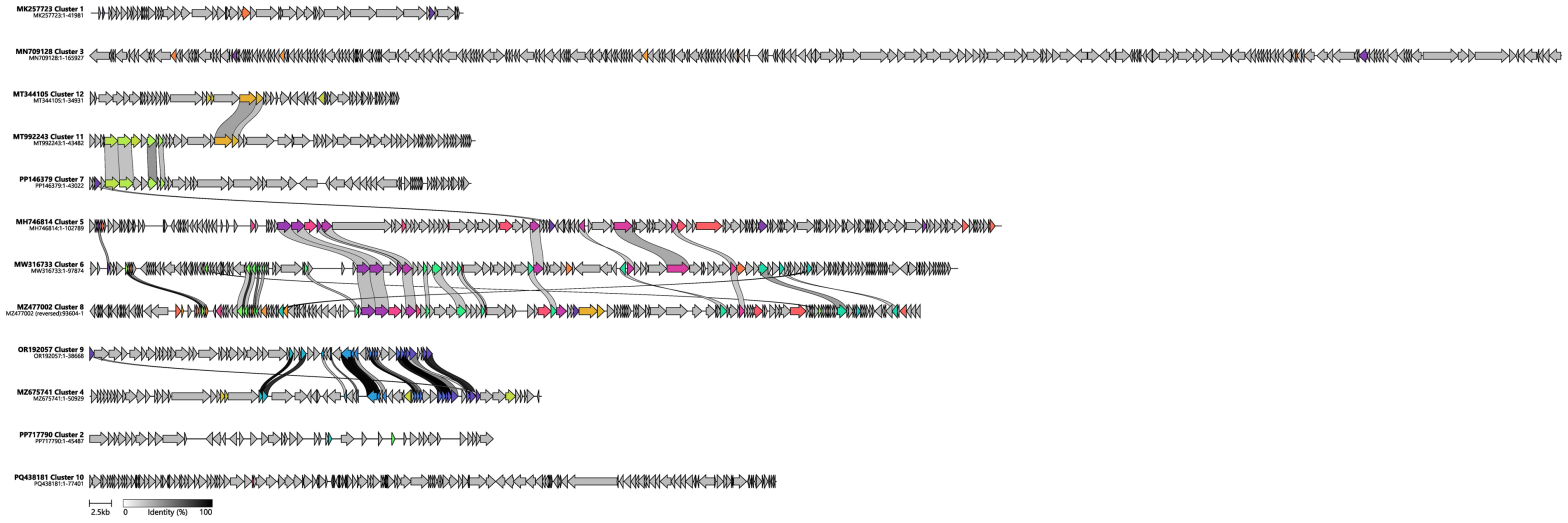
Between-cluster gene synteny of representative *Acinetobacter* phage genomes from 12 clusters. One randomly selected genome per PhamClust cluster was compared using Clinker to visualize shared homologous proteins across clusters. Genomes were ordered vertically by their overall syntenic similarity. Arrows represent predicted open reading frames, ribbon connectors link homologous proteins, with shading intensity proportional to amino-acid identity scaling from light gray (30% identity) to black (100% identity).

Next, we sought to validate the phylogenetic relationships inferred from marker-gene trees and investigate the conserved proteins within the related clusters. We performed a between-cluster synteny comparison using Clinker to potentially reveal shared mechanistic features shared between phylogenetically close lineages (Figure 3). For each of the 12 PhamClust clusters, a single representative genome was chosen at random. These 12 genomes were then analyzed collectively to detect homologous proteins and visualize conserved gene neighborhoods.

Aside from the closely related cluster pairs, most clusters share almost no amino-acid identity with one another. In the between-cluster map, most genes are connected only by light-gray ribbons indicating the 30% identity threshold or not linked at all, reflecting minimal detectable homology.

Clusters 11 and 12 uniquely share only two carbohydrate-active enzymes, an acetylxylan esterase and a Concanavalin A–like lectin/glucanase while the remainder of their proteomes are distinct. Similarly, Clusters 7 and 11 only share 4 proteins, large terminase subunit, portal protein and a major capsid protein as well as a hypothetical protein contrary to what the phylogenetic tree in Figure 2 suggests. Although no insertion sequence elements or transposase genes were identified, the presence of synteny between these shared genes indicates potential recombination between the respective clusters that could be driven by co-infections.

Clusters 5, 6, and 8 share extensive syntenic blocks encompassing both functional and structural modules and emerge from a common branch from the phylogenetic tree in Figure 2. This indicates that these three clusters could have evolved from a single common ancestor and undergone divergent evolution across the rest of their genomes. These clusters represent some of the three larger average genome sizes in our dataset, 102,985 bp (Cluster 5), 97,586 bp (Cluster 6), and 93,754 bp (Cluster 8), all with strictly lytic life cycles (Supplementary Table S2). Cluster 5 corresponds to the established genus *Saclayvirus*, whereas Clusters 6 and 8 lack current genus assignments. Their proximity to *Saclayvirus* suggests that they may share similar host-interaction strategies and genomic mechanisms, providing a starting point for future functional and taxonomic investigations.

Clusters 5 and 6 retains a set of core structural and replication genes: both encode a tail-fiber protein, a head decoration protein, and DNA polymerase in conserved gene order contexts. This shared repertoire suggests that these two lineages not only assemble virions with similar host-recognition and capsid-stabilization modules but also encode a homologous polymerase machinery for genome replication. Clusters 5 and 8 conserve a distinct set of proteins: a capsid maturation protease, tail fiber protein, endolysin, ribonuclease H like domain protein, ribonucleotide reduction large subunit, and a putative shock protein A. The presence of both replication (ribonucleotide reductase) and stress response (shock protein) enzymes suggests these phages may engage host machinery under distinct environmental conditions. All three clusters (5, 6, and 8) retain the core virion-assembly toolkit, the terminase large subunit, portal protein, tail-assembly chaperone, and HNH homing endonuclease, underscoring a common ancestral replication-and-packaging cassette.

Next, Clusters 4 and 9 also share a substantial proportion of their proteomes, potentially also evolving from a recent common ancestor, including methane monooxygenase, endolysin, an ATP-binding protein, CII protein, ribosomal protein, a carbohydrate-binding domain–containing protein, a transcriptional regulator, and several hypothetical proteins. Cluster 4 belongs to the known genus *Vieuvirus*. The shared protein repertoire suggests Cluster 9 may possess similar lysogenic control mechanisms, host receptor-binding strategies, and DNA replication processes.

### 3.4 Genome architecture and core proteome of novel Cluster 10

Given the identification of a novel cluster, designated Cluster 10, we sought to further examine its genomic architecture and gene synteny.

**Figure 4:**
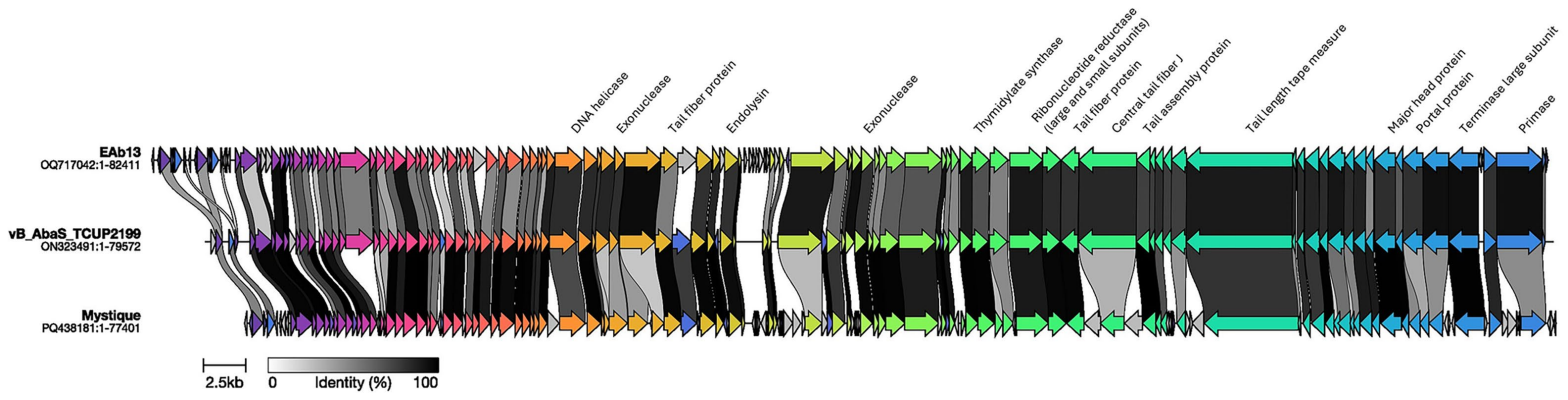
Between-cluster gene synteny map for Cluster 10 representatives. Three genomes, *Acinetobacter* phage vB_AbaS_TCUP2199 (ON323491:1–79572), EAb13 (OQ717042:1–82411), and Mystique (PQ438181:1–77401), were visualized with Clinker. Ribbons connect homologous proteins between genomes, and ribbon shading reflects amino-acid identity from light gray (30% identity) to black (100% identity).

Cluster 10, consisting of three broadly lytic *siphoviruses* as described by their respective publications, vB_AbaS_TCUP2199 (ON323491), EAb13 (OQ717042), and Mystique (PQ438181), exhibits well conserved shared modules across their genomic architecture (Figure 4). We found 81 core proteins shared across all three genomes that encoded 98, 126, and 154 predicted open reading frames (ORFs) respectively (Alseth et al., 2025; Mardiana et al., 2022; Margulieux et al., 2023). The majority of these conserved ORFs are annotated as hypothetical, reflecting the high proportion of novel genes in this cluster. This “genetic dark matter” is consistent with Cluster 10’s distant phylogenetic relationships with the other clusters (Figures 2, 3). Therefore, we expected many proteins found in these phages to be novel without much shared amino acid identity as compared to those encoded by those in more studied clusters.

Among the core proteins with functional annotations, we observed several enzymes central to nucleotide metabolism, such as a ribonucleotide reductase (large and small subunits), a primase, a DNA helicase, two exonucleases, and a thymidylate synthase enzyme. Structural components were also included, such as portal protein, large terminase subunit, major head protein, tail length tape-measure protein, tail assembly protein, two distinct tail-fiber proteins and a central tail-fiber J protein. In addition, an endolysin was identified as a core protein.

### 3.5 Proteomic conservation across clusters

Given the vast diversity of protein families observed across our *Acinetobacter* phage dataset, we next asked whether any truly universal “core” proteins exist. From our dataset of 30,824 protein sequences drawn from 250 complete *Acinetobacter* phage genomes, PhaMMseqs assembled a total of 4,133 gene phamilies (“phams”) spanning structural, functional, and uncharacterized proteins. The annotation of these phams can be found in Supplementary Table S3. Among these, 379 phams (9.2%) correspond to canonical virion-assembly components that were identified by keyword matches to capsid, tail, portal, baseplate, spike, sheath, tube, tape measure, connector, scaffold, decoration, vertex, hub, wedge, pin, or neck. A total of 842 phams (20.4%) carry putative enzymatic or regulatory functions such as DNA replication, transcriptional regulation and lysis. The nearly two-fold excess of functional over structural phams reflects the broad diversity of accessory proteins phages employ for host takeover and environmental adaptation. Of the total gene families identified, 2,912 phams (70.5%) were annotated as hypothetical proteins, highlighting the extent of unexplored “genetic dark matter” within phage proteome.

Remarkably, not a single gene family qualified as “core” (found in ≥99% of genomes) or “soft-core” (95–99%), indicating there is no universally conserved phage protein across all the *Acinetobacter* phages. Instead, only 96 phams (2.32%) fell into the “shell” category, defined as being present in 15–95% of genomes, representing conserved modules shared by multiple phages. The overwhelming majority (4,037; 97.7%) of phams resided in the “cloud,” occurring in fewer than 15% of genomes and were confined to single clusters or individual isolates.

Most phams are confined to a single cluster and rarely shared between 2 to 3 clusters. Identifying phams that could be found in four or more clusters could be prime candidates for illuminating key enzymatic and structural modules (Table 1). Such broadly conserved phams likely perform essential biological functions in *Acinetobacter* phages, having been repeatedly selected by evolution for efficiency or necessity. They could also uncover promising targets for phage engineering and effective antimicrobial agents.

**Table 1:** Phamilies conserved across four or more PhamClust clusters of *Acinetobacter* phages.

Pham	Function	Number of clusters	Clusters	Prevalence in clusters (%)
pham_1	Endolysin	5	1, 2, 5, 7, 8	1: 97.8%; 2: 46.5%; 5: 90.0%; 7: 80.0%; 8: 100.0%
pham_39	Hypothetical	5	2, 4, 6, 8, 9	2: 83.7%; 4: 86.7%; 6: 100.0%; 8: 50.0%; 9: 100.0%
pham_48	Endolysin	5	2, 3, 4, 5, 6	2: 16.3%; 3: 100.0%; 4: 10.0%; 5: 10.0%; 6: 100.0%
pham_44	DNA helicase	4	2, 4, 9, 12	2: 90.7%; 4: 63.3%; 9: 25.0%; 12: 100.0%
pham_60	Endolysin	4	2, 4, 7, 9	2: 37.2%; 4: 86.7%; 7: 20.0%; 9: 100.0%
pham_106	HNH homing endonuclease	4	2, 5, 6, 8	2: 48.8%; 5: 20.0%; 6: 66.7%; 8: 100.0%
pham_344	hypothetical	4	2, 4, 8, 9	2: 23.3%; 4: 46.7%; 8: 50.0%; 9: 50.0%
pham_651	hypothetical	4	1, 8, 11, 12	1: 1.1%; 8: 25.0%; 11: 100.0%; 12: 100.0%

Each row lists the phamily identifier, its annotated function, the number of clusters in which it appears, the specific cluster number, and the percentage of genomes within each cluster encoding that pham.

Interestingly, three distinct endolysin phams are broadly distributed: pham_1 endolysin and pham_48 endolysin appear in five clusters, cluster 1, 2, 5, 7, 8 and clusters 2, 3, 4, 5, 6, respectively, and pham_60 endolysin in four clusters, cluster 2, 4, 7, 9. Pham_44, a DNA helicase, and Pham_106, an HNH homing endonuclease, each recur in four phylogenetically distinct clusters—Pham_44 in Clusters 2, 4, 9, and 12, and Pham_106 in Clusters 2, 5, 6, and 8. Three phamilies remain uncharacterized, two occurring in four clusters (pham_344, pham_651) and one in five clusters (pham_39).

In summary, the conservation of these identified endolysins, DNA helicase, HNH homing endonuclease, and three hypothetical proteins across multiple, phylogenetically distinct *Acinetobacter* phage clusters could serve as potential entry points for phage modification and phage-derived antimicrobials.

### 3.6 Cluster specific gene phamilies

To uncover the unique molecular signatures that distinguish each PhamClust group, we identified cluster-specific phamilies that were highly prevalent (at least 90%) in the genomes within their respective clusters. By focusing on these cluster-specific phams, we aim to elucidate cluster-defining traits, such as specialized adsorption apparatus, niche-adapted enzymes, or regulatory factors. We examined only the first five identified clusters given that the rest of the clusters have a relatively smaller sample size of less than ten members, hence analyzing for cluster specific phamilies within them would not give us an accurate view of the cluster (Supplementary Table S4).

Cluster 1 comprise phages from *Daemvirus*, *Friunavirus*, *Pettyvirus*. They retain a full complement of DNA processing and structural phams. The universal presence of tail tubular protein A (pham_8) (UniProt, 2025), a hallmark structural component of short, noncontractile tails (Podoviridae | ICTV, 2025), correlates with these phages’ *Podoviridae*-like morphology. Likewise, the co-occurrence of multiple DNA-processing enzymes-5′-3′ exonucleases (pham_4, pham_7), endonuclease VII (pham_13), DNA helicase (pham_14), and a dedicated RNA polymerase (pham_9) reflects a conserved middle-stage replication program potentially unique to Cluster 1, underscoring its self-sufficient DNA replication machinery.

Cluster 2 phages form a distinct *Obolenskvirus* genus defined by a unique complement of contractile-tail assembly proteins, including baseplate hub (pham_72), tail sheath (pham_76), tail tube initiator (pham_78), tail tube protein (pham_91), tail completion factor (pham_79) and a unique tail-fiber protein (pham_94). These specialized tail assembly proteins could be adapted for host specificity, targeting a specific subset of *A. baumannii* hosts (Taslem Mourosi et al., 2022). Complementing this structural arsenal, Cluster 2 carries its own transcriptional machinery (pham_74 RNAP and pham_95 regulator) enabling immediate, host independent early gene expression and precise temporal control over the infection cycle. The universal retention of head related components, a major head protein (pham_85) and prohead core protease (pham_87) underscores a self-contained capsid assembly pathway preserved across the cluster.

Cluster 3 phages comprise phages from *Hadassahvirus*, *Lazarusvirus* and *Zedzedvirus* genera with some of the largest genomes in our dataset (approximately 166 kb in size). They encode multiple baseplate and tail fiber components including hub subunits (pham_125, pham_134), wedge modules (pham_130, pham_137), a hinge connector for long tail fibers (pham_127), and a tail sheath (pham_133) coupled to a tail tube (pham_126) indicative of a cluster specific adsorption device for host recognition and DNA injection. Alongside this structural core, they carry a self-contained capsid assembly pathway (pham_138 prohead core protein) and dedicated lytic machinery (pham_136 putative murein transglycosylase) for its specialized cell-wall degradation mechanism (Bieńkowska-Szewczyk and Taylor, 1980). Uniquely, Cluster 3 harbors metabolic and genome-processing modules, such as an aerobic NDP reductase (pham_131) to boost dNTP pools, a DNA helicase loader (pham_128) for replication initiation, and an endonuclease V (pham_135) for DNA repair or recombination.

Strikingly, the only conserved core pham of Cluster 4, *Vieuvirus*, is the tail-assembly chaperone (pham_361). Unlike other clusters with extensive suites of fixed structural or replication modules, Cluster 4 lacks conserved machinery.

Cluster 5 phages define the *Saclayvirus* genus and are distinguished by a self-contained nucleotide-biosynthesis toolkit, dual thymidylate synthases (pham_358, pham_494), a MazG-like pyrophosphatase (pham_492), ribonucleotide reductase (pham_497), dihydrofolate reductase (pham_518) and thymidylate kinase (pham_576) together with complementary replication enzymes (DNA helicase pham_480, dNMP kinase pham_491, GTP-binding protein pham_513). The self-contained nucleotide-biosynthesis and replication enzymes toolkit of Cluster 5 likely accelerate genome replication under nucleotide-limited conditions or stressful conditions (Yuan and Gao, 2017a). The unique tail apparatus is equally elaborate with a baseplate hub (pham_478), tape measure protein (pham_490) and dedicated tail assembly chaperone (pham_484), three distinct tail fibers (pham_470, pham_495, pham_530) and a short fiber module (pham_585). Given that *Saclayvirus* phages can infect *A. baumannii* strains with multiple distinct capsule types, this unique ensemble of tail proteins likely underlies their broad-host-range capability (Qin, 2025).

## 4 Discussion

### 4.1 Phages infecting *Acinetobacter* are deeply diverse and divergent

Phages infecting *Acinetobacter* likely originate from distinct evolutionary lineages that are deeply diverse and divergent, and possible polyphyletic in nature with inter-cluster proteomic similarity rarely exceeding 10% (Figure 1). Given the increasing number of clusters identified with greater sampling depth, we hypothesize that we have yet to reach rarefaction of phages and there are many more diverse cluster types to be discovered. Our lab has discovered novel *Acinetobacter* phages with no genomic similarity to the 12 clusters in this study, further supporting our hypothesis (unpublished data).

The low prevalence of shell pham in our database (conserved modules shared by multiple phages) as well as the lack of any core genes, further reflects the modular and mosaic nature of phage genomes, that could be driven by high rates of horizontal gene transfer, recombination, and functional specialization, which rapidly shuffle gene repertoires across lineages and ecological niches (Hatfull, 2008). Even among the relatively closer groups, such as Clusters 4 and 9 or Clusters 5, 6, and 8, the observed homology is subtle, indicating long-standing separation and minimal exchange of conserved modules.

This absence of universally conserved core genes is not unique to *Acinetobacter* phages but reflects a broader characteristic of phage genomics. Similar findings have been reported in jumbo phages (Magar et al., 2024) and *Streptococcus thermophilus* phages (Szymczak et al., 2019), where no single gene was conserved across all genomes. These observations collectively highlight the mosaic architecture and extensive genomic diversity that underpin phage genomics.

### 4.2 Strong within-cluster protein homology and conserved genomic lengths except cluster 4

Although *Acinetobacter* phage clusters are highly divergent from one another, they typically exhibit strong within-cluster protein homology and conserved genome length. Notably, Cluster 4 corresponding to *Vieuvirus* shares less conservation within its cluster. Their elevated variability likely reflects greater genomic plasticity and recombination found within temperate phages, consistent with Lai et al. (2024) showing higher rates of genetic exchange with bacterial chromosomes compared to virulent phages. The relative looseness of this cluster and the absence of conserved, cluster-specific proteins suggest that *Vieuvirus*, as currently defined by ICTV, may represent a highly variable lineage, or could potentially comprise multiple distinct genera rather than a single cohesive genus.

### 4.3 Cluster 3 presents as near-jumbo phages

Cluster 3 exhibits genomic and structural features characteristic of jumbo phages, suggesting a “jumbo-like” classification despite its genome size of ~166 kb falling below the conventional 200 kb threshold (Yuan and Gao, 2017a). The combination of specialized baseplate structures, self-contained replication machinery, and numerous accessory components aligns with the mechanisms of jumbo phages. The jumbo-like cluster harbors a distinctive set of accessory phamilies absent from other clusters, highlighting its unique functional repertoire. As jumbo phages are thought to arise through the stepwise acquisition of novel genetic material that expands both genome size and functional capacity (Yuan and Gao, 2017b), Cluster 3 may represent an intermediate lineage on a trajectory toward jumbo phage status over evolutionary time.

### 4.4 Novel lineage with broad spectrum lytic activity—Cluster 10

Cluster 10 constitutes a novel lineage with exceptionally broad host range likely due to its conserved multiple tail-fiber and tail-associated proteins, the key determinants of receptor binding and host specificity. *Acinetobacter* phage Mystique lysed 94/103 *A. baumannii* strains, *Acinetobacter* phage vB_AbaS_TCUP2199 infected 189/208 clinical isolates, and *Acinetobacter* phage EAb13 was active against 91/105 multidrug-resistant strains. Both vB_AbaS_TCUP2199 and Mystique phages were shown previously to be strictly lytic, consistent with our PhaBOX lifestyle predictions (Alseth et al., 2025; Mardiana et al., 2022; Margulieux et al., 2023). High-resolution structure is available only for Mystique revealing a *siphovirus*-like architecture. Cluster 10 phages conserving this repertoire of tail related proteins are potential models for rational engineering of broad-host-range therapeutics against drug-resistant *A. baumannii*. Next steps could include defining these tail proteins, solving their structures, and testing domain-swap variants to map sequence-function rules for adsorption across diverse clinical strains.

### 4.5 Tail associated proteins are highly diverse but remain cluster-specific

In all 12 clusters, the most variable gene families across clusters encode tail related proteins and each cluster maintains its own unique repertoire of tail related proteins. Especially in the context of Cluster 3 and 5, our two largest genome clusters, they contain multiple conserved fiber families, alongside diverse tail chaperones, sheath, tube, and assembly factors. The observed variation in tail proteins suggests that not all tail components are involved in host recognition, supporting the notion that primarily the receptor-binding proteins mediate host specificity (Le et al., 2013). Future studies could investigate these cluster-specific tail proteins, as their high degree of conservation within clusters may serve as useful markers for distinguishing phage genera or clusters.

### 4.6 Horizontal transfer between clusters: two carbohydrate-active enzymes in Clusters 11 and 12 for phage-host binding

Among the highly divergent Clusters 11 and 12, we identified two carbohydrate-active enzymes, Acetylxylan esterase and a Concanavalin A-like lectin/glucanase superfamily protein, whose functions in capsule recognition and degradation may provide novel insights into the mechanisms of viral attachment to bacterial hosts (Peters et al., 2024; Pickard et al., 2010). This convergent adaptation to *Acinetobacter*’s carbohydrate-rich surface raises the possibility of overlapping host ranges, though neither original study assessed cross-cluster host susceptibility (Badawy et al., 2020; Pehde et al., 2021). Targeted host-range assays of representative Cluster 11 and 12 phages will be essential to test this hypothesis potentially shedding light on *Acinetobacter* phage-host binding and entry.

### 4.7 Gaps in taxonomical annotations

Taxonomical annotations using PhaBOX revealed substantial gaps in current phage taxonomy, with six of the twelve clusters lacking ICTV-recognized genus assignments. While PhaBOX classified the remaining clusters into established genera, these unassigned lineages underscore the limitations of existing frameworks and the need to expand reference databases to capture *Acinetobacter* phage diversity. For the ICTV assigned genera, the proteomic-based clustering approach was largely concordant with their taxonomy. However, this approach provides greater resolution, revealing subcluster structure within established genera. For example, Cluster 4, currently classified as *Vieuvirus*, displays heterogeneous clustering patterns and evidence of potential subclusters, suggesting that *Vieuvirus* may comprise multiple distinct genera. This approach can also capture clusters with only a few members that have not yet been assigned a genus by ICTV. By comparing these small clusters to related or homologous clusters, we can gain insights into their potential evolutionary lineage, functional modules, and shared mechanisms, highlighting relationships that are not apparent from current ICTV classifications like what we did for Clusters 4 and 9 as well as Cluster 5, 6, and 8.

### 4.8 Markers are not discriminatory for subcluster groupings

The use of concatenated terminase large subunit and portal protein sequences closely recapitulates full-proteome cluster groupings, validating their potential as accurate phylogenetic markers (Figure 2). This suggests a simpler, faster route to *Acinetobacter* phage classification analogous to 16S rRNA typing in bacteria enabling rapid clustering and characterization without full-genome analyses (Janda and Abbott, 2007). However, while the terminase large subunit and portal protein are effective for assigning phages to their respective clusters, their phylogenetic signal alone cannot accurately resolve how closely related those clusters are. As seen with Clusters 7 and 11, which appear as sister clades yet share only four genes by synteny, as well as Cluster 4 and 9 which the phylogenetic tree fails to identify as more related clusters, whole-proteome analyses are required to capture true evolutionary relatedness between clusters.

### 4.9 Applications: candidates for phage engineering

Our analysis also identified three conserved enzymes, endolysins, a DNA helicase, and an HNH homing endonuclease, present across multiple, phylogenetically distinct *Acinetobacter* phage clusters indicates strong evolutionary conservation, underscoring their potential as robust, naturally optimized candidates for phage engineering. As the findings reported here are derived solely from computational analyses, additional downstream experimental validation is required. Nevertheless, this framework serves to prioritize and refine a focused set of phage lineages, gene families, and functional modules that represent particularly promising candidates for targeted wet-lab investigation.

Endolysins target conserved peptidoglycan linkages, enabling broad lytic activity and making them strong candidates for lysin-based antimicrobials (Abdelrahman et al., 2021). The DNA helicase appears well adapted for efficient phage replication (Lo and Gao, 2021; Brok-Volchanskaya et al., 2008), while the HNH endonuclease likely enhances genome maturation, packaging, and recombination (Zhang et al., 2017). Their cross-cluster conservation suggests strong evolutionary selection for these functions, marking them as “nature-approved” modules for engineering phages with improved replication, adaptability, and lytic potency against diverse *A. baumannii* strains. Future work should benchmark pham_1, pham_48, and pham_60 endolysins head-to-head for bactericidal potency and breadth across diverse *Acinetobacter* strain panels to test whether their cross-cluster conservation corresponds to broad and robust killing activity.

## 5 Conclusion

This study has utilized a proteome-based framework for *Acinetobacter* phage diversity, resolving 12 clusters and 14 singletons while highlighting key translational opportunities. Broadly conserved enzymes, particularly endolysins, emerged as robust candidates for phage-derived antimicrobials. Cluster 10 represents a novel, broad-host-range lineage with strong therapeutic promise, driven by its conserved tail-fiber repertoire. At the same time, half of the clusters remain outside current ICTV taxonomy, underscoring the urgent need to expand phage classification systems to keep pace with new discoveries.

Together, these findings provide potential leads for phage and phage-based therapies while underscoring our current gaps in phage genomics and taxonomy.

## Data Availability

The datasets presented in this study can be found in online repositories. The names of the repository/repositories and accession number(s) can be found at: https://figshare.com/, https://doi.org/10.6084/m9.figshare.30295204.v1.
