# Supplementary material for: Genomic landscape of complete Acinetobacter phages: clustering, core-shell genes, and synteny insights
Source: Front Microbiol. 2026 Jan 28;17:1720092. doi: 10.3389/fmicb.2026.1720092 (PMC12893723; doi:10.3389/fmicb.2026.1720092)
Supplement: Supplementary file 5 [file Data_Sheet_1.docx]

#!/usr/bin/env python3
 """
 Split a multi-FASTA file into one FASTA per genome (organism).
 Each genome name is extracted from text inside [square brackets] in the header line.
 """

from pathlib import Path
 import re
 from collections import defaultdict

# === YOUR FILE PATHS remember to input ===
 input_fasta = Path("input your file path")
 output_dir = Path("input your output path")
 output_dir.mkdir(parents=True, exist_ok=True)

# === FUNCTIONS ===
 def sanitize_filename(name: str) -> str:
 """Clean organism name for safe filenames."""
 name = name.strip().replace(" ", "_")
 return re.sub(r"[^A-Za-z0-9._-]", "_", name)

def wrap_seq(seq: str, width: int = 60) -> str:
 """Wrap sequence lines to a fixed width."""
 return "\n".join(seq[i:i+width] for i in range(0, len(seq), width))

# === MAIN PARSING ===
 groups = defaultdict(list)
 org_regex = re.compile(r"\[([^\]]+)\]")

current_header = None
 current_seq = []
 current_org = "Unknown_Organism"

with input_fasta.open("r") as f:
 for line in f:
 line = line.strip()
 if not line:
 continue
 if line.startswith(">"):
 # Save previous record
 if current_header:
 seq = "".join(current_seq)
 groups[current_org].append((current_header, seq))
 # Start new record
 current_header = line
 match = org_regex.search(line)
 current_org = match.group(1) if match else "Unknown_Organism"
 current_seq = []
 else:
 current_seq.append(line)

# Save the final record
 if current_header:
 seq = "".join(current_seq)
 groups[current_org].append((current_header, seq))

# === WRITE PER-GENOME FASTA FILES ===
 for org, records in groups.items():
 safe_name = sanitize_filename(org)
 out_path = output_dir / f"{safe_name}.fasta"
 with out_path.open("w") as out:
 for header, seq in records:
 out.write(f"{header}\n{wrap_seq(seq)}\n")

print(f"‚úÖ Created {len(groups)} FASTA files in {output_dir}")
